# Ensemble Machine Learning Approaches Predict Survival in Lower-Grade Glioma Based on Glycosphingolipid Gene Expression and Metabolic Modeling

**DOI:** 10.34133/csbj.0143

**Published:** 2026-06-26

**Authors:** Jack W. J. Welland, Janet E. Deane

**Affiliations:** Cambridge Institute for Medical Research, Department of Clinical Neuroscience, University of Cambridge, Cambridge, UK.

## Abstract

Glycosphingolipids (GSLs) are essential components of biological membranes with important roles in cell signaling. Disrupted GSL metabolism is associated with malignancy across a range of cancers, with different GSLs implicated in distinct tumors. GSLs have potential mechanistic roles in cancer; however, their functions in lower-grade gliomas (LGGs) remain poorly understood. We present ensemble machine learning approaches using transcriptomic data from LGG, combined with GSL-specific metabolic simulations, to predict survival outcomes. The ensemble approach demonstrates effective risk stratification for LGG patients based on GSL synthetic enzyme expression. Pathway analysis of model-derived risk groups identified correlations with GSL-modulated pathways including cell motility, division, and Wnt signaling in LGG pathology. Given the strong performance of machine learning approaches to predict survival outcomes and that GSLs are shed into the tumor microenvironment, GSL-based diagnostics and prognostics may prove to be clinically beneficial upon future experimental validation. A Python package enabling GSL-specific metabolic modeling and risk prediction from RNA sequencing data is provided.

## Graphical Abstract

**Figure:**
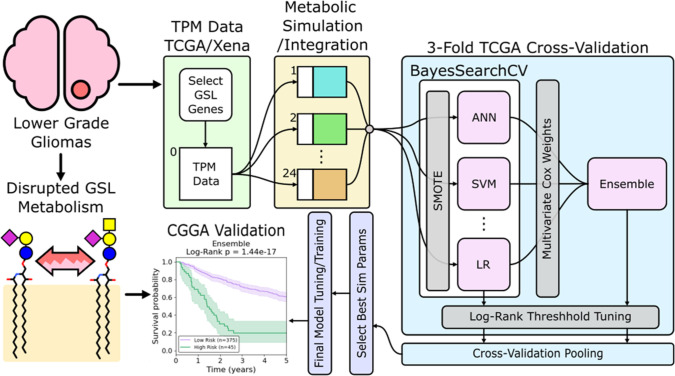

## Introduction

Lower-grade gliomas (LGGs) are a type of primary brain tumor that can develop in both children and adults and can severely impact neurological function [1]. Gliomas possess substantial molecular and clinical heterogeneity, which presents a severe challenge for precise diagnosis and treatment [2–8]. Therefore, detailed molecular profiling and identification of key markers is critical for improving patient stratification and guiding diagnosis and prognosis [9]. In large transcriptomic resources such as The Cancer Genome Atlas (TCGA), LGG is used to describe diffuse gliomas of World Health Organization (WHO) grades II and III, encompassing astrocytomas and oligodendrogliomas [10,11]. However, in the updated 2021 WHO classification, gliomas are defined primarily by molecular features, particularly isocitrate dehydrogenase (IDH) mutation status, with entities such as IDH-mutant astrocytoma (grades 2 to 4) and IDH wild-type glioblastoma (GBM) considered biologically distinct tumor types [12,13]. In this study, we adopt the TCGA-based LGG designation for consistency with the transcriptomic datasets used while acknowledging that this grouping does not directly map onto current molecular classifications.

**Fig. 1.**
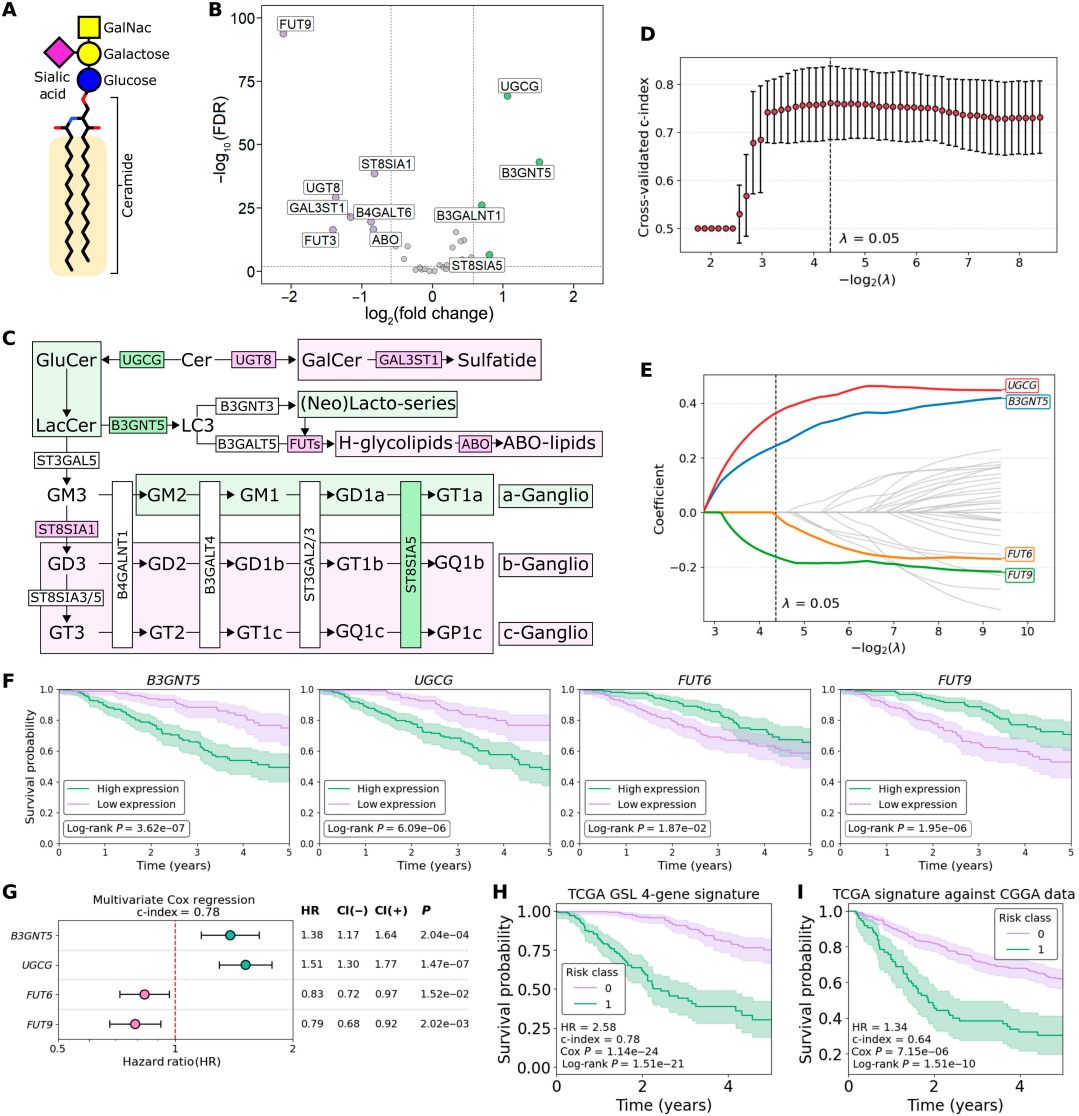
Analysis of glycosphingolipid (GSL) differential gene expression in glioblastoma multiforme (GBM) and lower-grade glioma (LGG). (A) Schematic diagram of a model GSL, the ganglioside GM2. The ceramide tail and individual headgroup sugars are labeled. (B) Differential expression analysis of The Cancer Genome Atlas (TCGA) RNA sequencing (RNA-seq) data for GBM against LGG. Significantly up-regulated genes in GBM are highlighted in green, while significantly down-regulated genes are highlighted in purple. A >1.5-fold change and *P* < 0.01 thresholds were used to determine significance. *P* value calculated as standard in DESEQ2 (Wald test) and false discovery rate (FDR) calculated through Benjamini–Hochberg adjustment. (C) Schematic diagram of part of the GSL synthetic pathway, highlighting changes in gene expression in GBM *vs* LGG in corresponding colors. Potentially impacted lipids/lipid series are also highlighted to align with the differential expression data. (D) Cross-validated lasso regression against expression values for 34 genes of GCL synthesis from TCGA LGG records, scoring against Cox proportional hazards multivariate concordance index (c-index). The optimum *λ* parameter (maximizing the c-index) is highlighted by a dashed line. (E) Cox coefficients from multivariate Cox regression experiments against TCGA gene expression data for LGG patients. The optimal lambda of 0.05 is shown with a dashed line, with nonzero coefficient genes at this *λ* highlighted in color. (F) Kaplan–Meier (KM) analysis of the 4 genes identified in panel (E). High- and low-expression groups were determined via stratification around the median expression value for each gene, using TCGA LGG data. Log-rank test used to calculate *P* values. (G) Multivariate Cox regression of TCGA LGG data performed against the 4 selected genes, showing hazard ratios in the plot window, with 1.0 marked as a red line. *P* values were calculated as part of the Cox analysis via the Wald test. (H) KM analysis of TCGA risk stratification on the GSL risk score with a log-rank test tuned decision threshold. (I) KM analysis of CGGA LGG data stratified by a risk score derived from the multivariate Cox regression coefficients for each of the 4 genes in panel (G), based on TCGA data. In (H) and (I), both the log-rank test *P* and the Cox proportional hazards Wald test *P* values are reported.

Glycosphingolipids (GSLs) are bioactive, glycosylated lipids that are aberrantly expressed in several cancers and play crucial roles in brain development and brain cell function [14,15]. GSLs possess diverse glycosylated headgroups ranging from single sugars to complex branched glycan chains (Fig. 1A) and are enriched in the outer leaflet of the plasma membrane where they engage in cell–cell interactions, influence cell signaling, and modulate immune responses [16–18]. In cancer, changes to GSL profiles contribute to cell proliferation, invasion, and epithelial-to-mesenchymal transition promoting metastasis [19–21]. GSLs can regulate signal transduction by interacting with growth factor receptors influencing cell growth and apoptosis [15]. GSLs are known tumor-associated antigens and are used as diagnostic markers in several cancers [20]. In the context of gliomas, it has been previously shown that the key (neo)lacto-series gatekeeper enzyme B3GNT5 is associated with poor prognosis in GBM, with gene expression significantly associated with glioma stem cells and *B3GNT5* knockdown resulting in decreased neurosphere formation [22]. Interestingly, similar behavior has been observed in prostate cancer, where again *B3GNT5* knockdown resulted in decreased cancer stem cell sphere formation [23].

GSLs are synthesized via a series of stepwise additions of glycan moieties by catalytic enzymes including glycosyltransferases, sialyltransferases, and fucosyltransferases [24–26]. The combined gene expression and activity of these enzymes produce the different GSL repertoires present in a given cell type. This GSL repertoire changes during development and is cell type specific [27]. The restricted expression of some GSL subtypes, such as GD2 and fucosylated GSLs, has made them ideal for targeted therapies [15]. Furthermore, inhibition of GSL synthesis can impair tumor growth and induce apoptosis [28].

Although individual GSL synthetic enzymes have been associated with specific cancers, there has been very limited exploration of how the broader family of synthetic genes are expressed in cancers and there has been no incorporation of such data into metabolic models for the prediction of the altered overall GSL repertoire. Advancements in machine learning strategies and metabolic modeling pipelines, combined with publicly available, highly curated transcriptomics datasets, are enabling new discoveries in cancer research [29]. Here, we explore the role of GSL synthetic enzyme gene expression in LGGs and identify that specific changes in 3 key genes are highly predictive for poor LGG prognosis. We sought to investigate the application of machine learning approaches against a broader suite of GSL biosynthetic enzymes to provide a more general description of GSL-related changes. We developed a risk score based on GSL synthetic enzyme expression utilizing an ensemble machine learning approach. This score can be applied to patients of LGG and can serve as a general indication of GSL-perturbation-associated severity. Furthermore, the known functional roles of GSLs in altered cell signaling may inform potential future therapeutic avenues targeting GSL-modulated pathways or GSL synthesis directly.

## Results and Discussion

### GSL synthetic enzymes are prognostic indicators in LGG as well as up-regulated in GBM

The first approach taken to investigate GSL metabolism as a candidate for risk modeling in LGG was to perform differential expression analysis between the gene expression profiles of LGG patients and the more severe GBM patients in the TCGA. Eleven key genes of GSL biosynthesis were significantly differentially expressed between the 2 cancers (*P* < 0.01, >1.5 fold change). Genes that were down-regulated in GBM compared to LGG corresponded to the fucosyltransferases FUT9 and FUT3, the galactosylceramide synthase UGT8, the sulfatide synthase GAL3ST1, one of the lactosylceramide (LacCer) synthases B4GALT6, histo-blood group ABO system transferase ABO, and the sialyltransferase ST8SIA1. Those that were instead up-regulated were the LC3 synthase/(neo)lacto-series gatekeeper B3GNT5, the glucosylceramide (GluCer) synthase UGCG, the sialyltransferase ST8SIA5, and the globotriaosylceramide synthase B3GALNT1 (Fig. 1B).

To better interpret the potential implications of these expression changes, they were mapped onto a reduced schema of GSL metabolism (Fig. 1C). The core unit of a GSL is the ceramide backbone, which can be initially modified by the addition of either a glucose via UGCG or alternatively a galactose via UGT8. This represents the first major metabolic bifurcation in GSL synthesis. The observed down-regulation of *UGT8*, combined with the up-regulation of *UGCG*, suggests that the most apparent of the expression-inferred changes to GSL metabolism would be an expected shift toward GluCer-based lipids, away from galactosylceramide-based lipids in GBM when compared to LGG.

Downstream of GluCer, there are further gene expression changes in GBM that have the potential to redirect GSL metabolism to alter the overall GSL repertoire of GBM versus LGG. The increase in *B3GNT5* expression, alongside the decrease in fucosyltransferase (*FUT9*/*FUT3*) and *ABO* expression, could suggest a prioritization of (neo)lacto-series lipids over the H- and ABO-glycolipids. Weaker inferences can be made for the gangliosides, where decreased *ST8SIA1* expression, coupled to increased *ST8SIA5* expression, could imply a push toward the more complex a-series gangliosides.

Following the differential expression analysis, to identify LGG-specific risk factors, Cox regression and Kaplan–Meier (KM) analyses were performed on TCGA LGG data. Elastic net lasso regression was tuned under cross-validation, identifying *λ* = 0.5 to maximize the concordance index (c-index) (Fig. 1D). The subsequent Cox regression identified 4 GSL synthetic genes with nonzero coefficients at *λ* = 0.5 out of the 34 genes included in the analysis. These genes were *UGCG*, *B3GNT5*, *FUT6*, and *FUT9* (Fig. 1E). Interestingly, those with positive coefficients, *UGCG* and *B3GNT5* in the LGG analysis, are also up-regulated in GBM when compared to LGG, while *FUT9’*s negative coefficient aligns with its down-regulation in GBM. This observation, that the genes that are implicated in poor prognosis within LGG are the same as those that are up-regulated in more severe GBM, strongly suggests a role for GSL metabolism in disease severity. KM curves for the 4 identified genes are shown in Fig. 1F, with risk groups separated around median expression.

To generate a 4-gene risk signature, multivariate Cox regression was performed on TCGA data (Fig. 1G). The 4-gene score was defined by the coefficient-weighted sum of expression values for each gene (Eq. (1)). The TCGA-trained coefficients were used to calculate risk scores for LGG data from a completely independent validation set, the Chinese Glioma Genome Atlas (CGGA). The risk group threshold was this time tuned on TCGA data based on maximizing the log-rank test statistic. The resulting KM analysis showed significant risk group stratification against the CGGA data (log-rank *P* = 1.5e−10). Univariate Cox analysis of the continuous risk score yielded a significant result against both the TCGA data and the CGGA validation data, with hazard ratios of 2.58 (*P* = 1.1 × 10^−24^, Fig. 1H) and 1.34 (*P* = 7.2 × 10^−6^, Fig. 1I). This result is comparable to similar analysis for LGG previously performed on fatty acid metabolism against TCGA data, yielding a 4-gene risk signature with a hazard ratio of 1.57 (*P* < 0.01) [30].

### Computational modeling of GSL metabolism with pyGSLModel as a feature engineering strategy for survival prediction

The GSL repertoire of a cell is not solely determined by the level of gene expression of the synthetic enzymes. The availability of substrates and enzyme–substrate preferences will contribute to the final GSL composition. Changes in gatekeeper/branchpoint enzyme expression, such as *UGCG*, *UGT8*, and *B3GNT5*, might be expected to result in substantial shifts in the GSL composition of a cell, but multiple smaller changes may also have the potential to shift GSL repertoires in complex and difficult-to-predict ways. With this in mind, despite the promising performance of the 4-gene GSL signature for survival modeling, metabolic simulation was utilized as a feature engineering approach to buttress model training.

We developed a Python package (pyGSLModel) to implement constraint-based metabolic simulations built on top of COBRApy [31], pyfastcore [32], and iMATpy [33]. The Integrative Metabolic Analysis Tool (iMAT) method was used to perform patient-specific simulations of GSL metabolism based on their GSL synthetic enzyme expression data using a small-scale metabolic model of GSL synthesis pruned from the HUMAN-GEM model [34] in pyGSLModel, preserving the core reactions of metabolism and reactions of GSL synthesis. The iMAT methodology for metabolic simulations involves the incorporation of gene expression weights from transcriptomic datasets alongside a metabolic model, in this case the GSL biosynthetic schema, and searches for a feasible flux distribution that maximizes the sum of high-expressing reactions where the flux is greater than a target threshold and low-expressing reactions where the flux is less than a target threshold. This approach enabled the incorporation of relationship information between GSL synthetic genes based on their relative positions within the metabolic network, with this information, in principle, represented in the corresponding reaction fluxes for a patient. This is particularly powerful for tackling the complex nature of GSL metabolism, where many GSL biosynthetic enzymes have multiple different substrates and products. The modeling approach utilized here has not been experimentally validated for GSL biosynthesis and should therefore be treated with caution, particularly relating to any biological interpretations based on simulated fluxes. The use case of this method explored here is to enrich GSL synthetic enzyme transcriptomic data through metabolic simulations that can incorporate the GSL biosynthetic schema. As this approach is not fundamentally grounded in experimental metabolomic/lipidomic data, the generalizability to other modeling or machine learning problems is limited, with parameters needing to be retuned for the specific problem at hand.

**Fig. 2.**
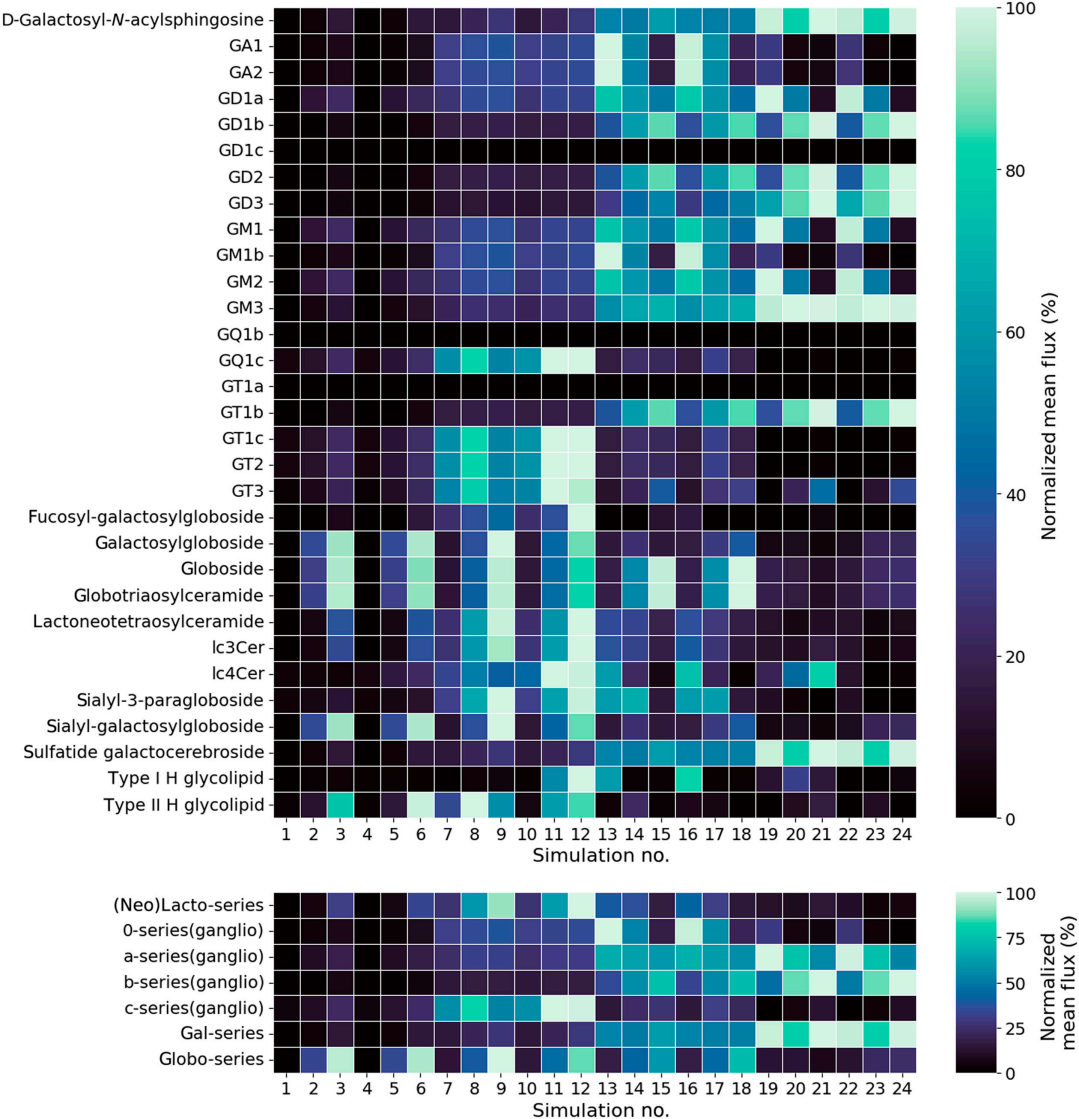
Glycosphingolipid (GSL) metabolic flux analysis. Top panel: Normalized mean simulated fluxes across all The Cancer Genome Atlas (TCGA) lower-grade glioma (LGG) patients are shown as a heatmap (colored from black through blue to light green) for each GSL species (rows) across each set of simulation parameters (columns and detailed in Table 1). Lower panel: Simulated fluxes across distinct GSL series were summed to give the total normalized mean flux per lipid series. Colored and displayed as in the top panel.

Transcriptomic integrative metabolic simulations were performed for TCGA and CGGA LGG data for genes of GSL synthesis across 24 different sets of iMAT input parameters (Table 1). Considerable variation in output can be seen across iMAT parameters (Fig. 2), demonstrating the need to trial different parameters and evaluate their impacts on model performance empirically. Generally, a shift away from globo-, (neo)lacto-, and ganglio c-series toward gal-series and a/b-series gangliosides was observed when increasing the epsilon (the minimum flux required for a reaction to be considered “active”) and threshold (the flux limit above which a lowly expressed reaction is penalized for being “active”) values. These 2 parameters both contribute to the objective function for the simulation that seeks to maximize the consistency between the data and the model. This parameter sensitivity highlights the need for caution when trying to make biological inferences based on these kinds of metabolic simulation, despite their potential empirical benefit for modeling tasks.

**Table 1.** Set of parameters trialed for iMAT integration

Simulation no.	Upper quantile (%)	Lower quantile (%)	Epsilon	Threshold
1	10	90	1	0.1
2	20	80	1	0.1
3	30	70	1	0.1
4	10	90	1	0.5
5	20	80	1	0.5
6	30	70	1	0.5
7	10	90	10	1
8	20	80	10	1
9	30	70	10	1
10	10	90	10	5
11	20	80	10	5
12	30	70	10	5
13	10	90	50	5
14	20	80	50	5
15	30	70	50	5
16	10	90	50	25
17	20	80	50	25
18	30	70	50	25
19	10	90	100	10
20	20	80	100	10
21	30	70	100	10
22	10	90	100	50
23	20	80	100	50
24	30	70	100	50

iMAT, Integrative Metabolic Analysis Tool

**Fig. 3.**
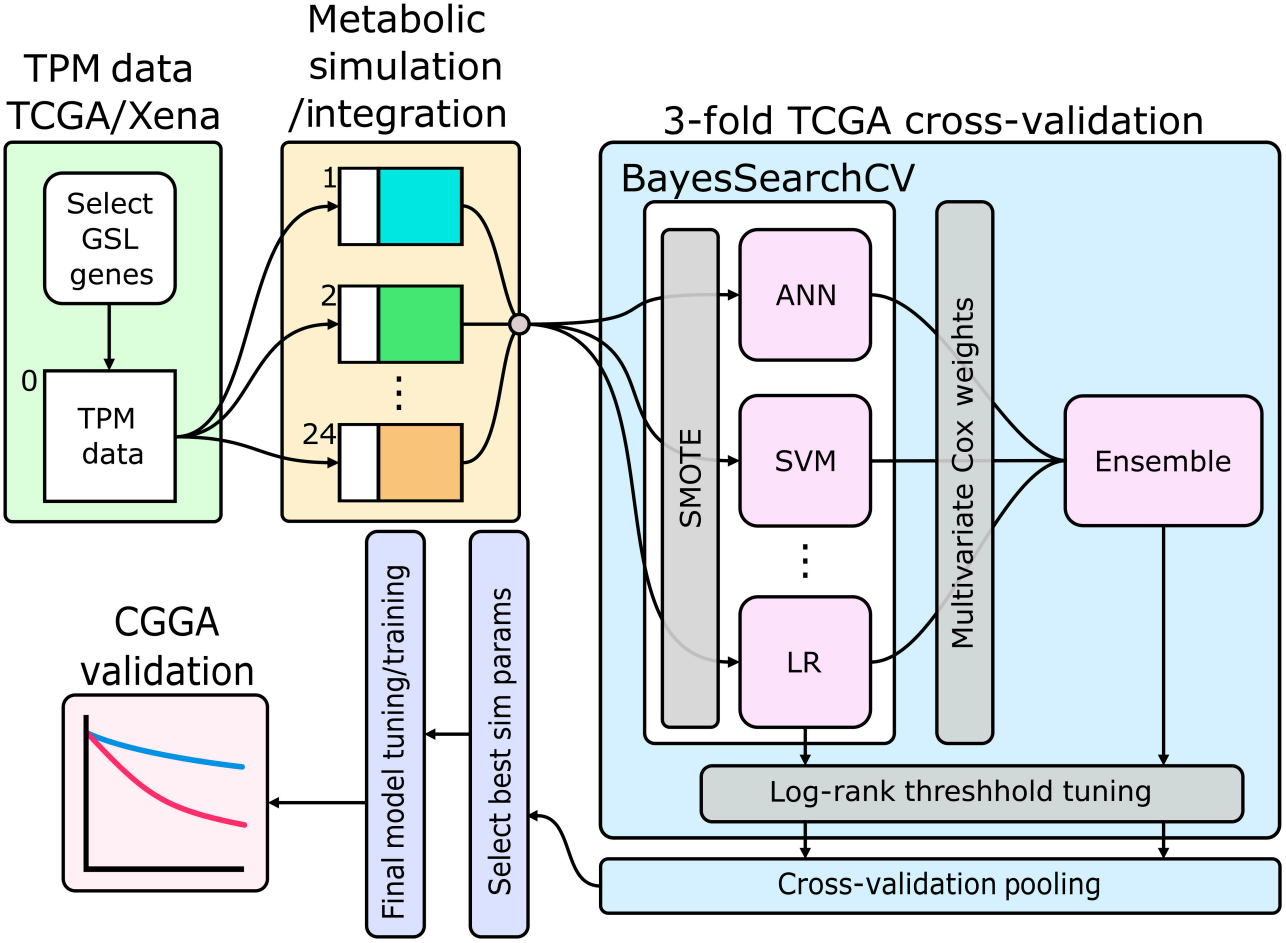
Schematic of the workflow of this study. The green box shows glycosphingolipid (GSL) synthetic gene transcripts per million (TPM) expression data accession from The Cancer Genome Atlas (TCGA) via Xena. The yellow box shows the integration of simulated metabolic data to append to the expression dataset. The light blue box shows the nested cross-validation (CV) machine learning approach. Data were subjected to the synthetic minority oversampling technique (SMOTE) to deal with class imbalance before training 5 different models to predict overall survival: artificial neural network (ANN), support vector machine (SVM), random forest (RF), XGBoost (XGB), and logistic regression (LR). Model output probabilities were then combined to generate an ensemble model based on multivariate Cox coefficients for each model. Threshold tuning was performed, and CV results were pooled for analysis. The dark blue box shows final model training and cross-validated tuning trained on all TCGA data, using metabolic simulation parameters identified in the nested CV experiment. The pink box denotes final model validation against completely independent lower-grade glioma (LGG) data from the CGGA.

Separate datasets were prepared corresponding to each different parameter set for model training, in which the simulated metabolic fluxes for GSL synthetic reactions were appended onto GSL synthetic enzyme expression data. This resulted in 24 separate datasets to be trialed in subsequent model development (Fig. 3).

### Ensemble machine learning approaches effectively model survival based on GSL synthesis

To generate a GSL risk score that captures patterns of change across GSL synthesis, rather than traditional gene signatures such as demonstrated above (Fig. 1H), an ensemble machine learning approach was implemented. This method allows us to utilize the full GSL synthetic gene expression data available, alongside the appended metabolic simulation reaction fluxes. An ensemble approach was chosen, rather than focusing on any one specific model, to offer increased robustness in final score calculation and risk stratification while also enabling the incorporation of time-to-event information through the multivariate Cox weighting of base models. Model performance was evaluated using complementary survival and classification metrics, including c-index, Cox-regression-derived hazard ratios, KM risk stratification with log-rank testing, and time-dependent receiver operating characteristic–area under the curve (ROC–AUC) analysis.

Five different machine learning models were trained, a support vector machine (SVM), a random forest (RF) model, an XGBoost (XGB) model, a logistic regression (LR) model, and an artificial neural network (ANN). Each model was trained separately on each of the 24 prepared datasets for each set of simulation parameters to predict overall survival. The final features included the gene expression data for the GSL synthetic enzymes (listed in Methods) alongside the simulated metabolic fluxes for key GSLs (listed in Methods) and total fluxes through GSL series. The data underwent *z*-score normalization, and the synthetic minority oversampling technique (SMOTE) was used to address class imbalance. A 2-step approach was utilized for model training in order to facilitate the tuning of iMAT metabolic simulation parameters without leading to data leakage before training the final model. In the first step, the models were trained and validated using only TCGA data under nested cross-validation with Bayesian hyperparameter optimization performed in the inner cross-validation folds to prevent data leakage. The ensemble model was engineered by performing multivariate Cox regression against the 5 trained models and subsequently weighting their probabilities by the Cox coefficients before calculating the weighted sum (Eq. (2)). This approach enables a survival-specific method for model weighting, incorporating survival time information as well as survival classification to produce the ensemble score. The resulting models were evaluated against the pooled cross-validation sets. Model thresholds were tuned based on identifying probability/ensemble score thresholds that maximized the log-rank test statistic. This approach is outlined in Fig. 3.

This process was repeated 5 times at different random states. The best-performing set of iMAT metabolic simulation parameters across repeats was determined by average c-index, as they provided a survival relevant description of performance. This parameter set was taken forward for final model training. iMAT simulation parameters of upper quantile = 30%, lower quantile = 70%, *ε* = 100, and threshold = 10 achieved the best performance, with an average c-index of 0.77. This corresponded to parameter set 21 (Table 1). This nested cross-validation approach prevented data leakage through the hyperparameter optimization or iMAT simulation optimization steps as only TCGA data were used in this process. The resulting iMAT parameters chosen from this process can then be taken forward for full training on TCGA data and evaluation on completely independent CGGA data.

The final models were trained using all TCGA data and underwent cross-validated Bayesian hyperparameter optimization. The resulting model was validated against the independent CGGA dataset, which had not been utilized at any point previously. Cox coefficients for ensemble weighting and the risk classification threshold were defined using only the training TCGA data to avoid data leakage. Identical genes of GSL synthesis were used and underwent metabolic simulations as per parameter set 21, which was selected based only on TCGA data.

**Fig. 4.**
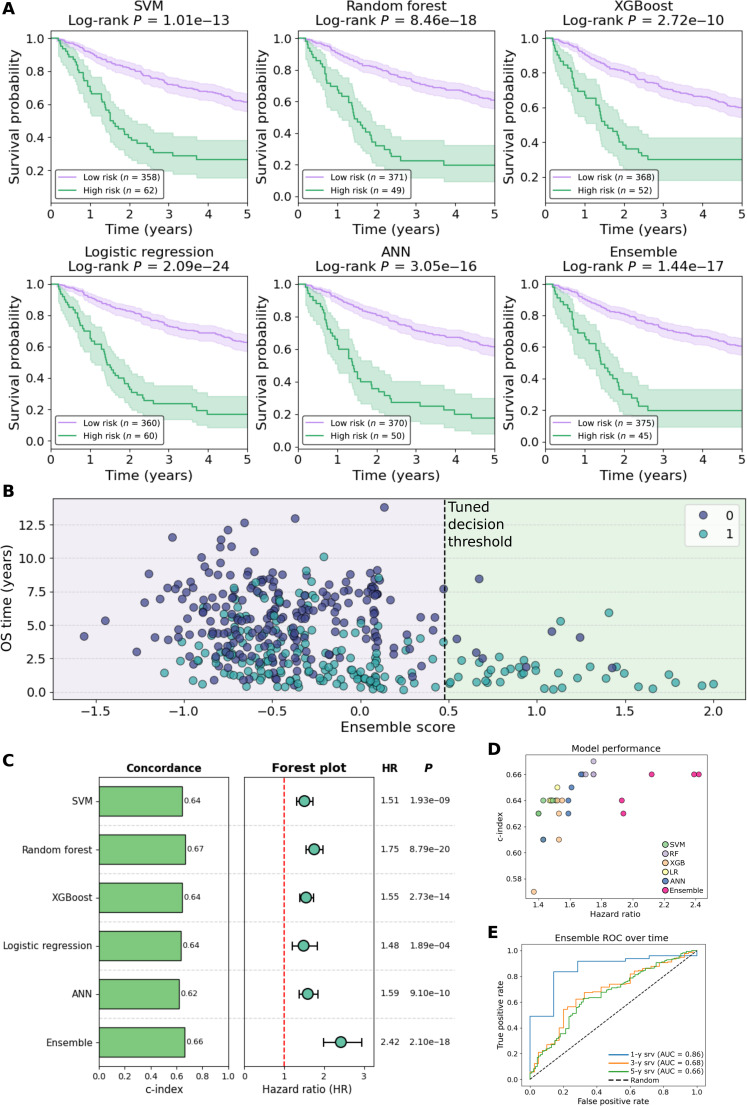
Patient stratification with ensemble machine learning (ML). (A) Kaplan–Meier (KM) survival curves showing stratification between ML-model-defined risk groups for The Cancer Genome Atlas (CGGA) data (models trained on The Cancer Genome Atlas [TCGA] data exclusively). Low-risk predictions are in purple, while high-risk ones are in green. Log-rank test *P* values are reported. (B) Overall survival (OS) time plotted against ensemble model scores. (C) Output performance statistics for each ML approach for the representative random state. The *P* value was calculated as part of the univariate Cox regression analysis, which utilizes the Wald test. (D) Model performance plot of the concordance index (c-index) against the hazard ratio. Each random state replicate for each model is plotted with colors separating the different ML approaches. (E) Receiver operating characteristic curve for a representative ensemble model (consistent with panel (A)). Data are separated by time intervals, showing curves for <1-year, <3-year, and <5-year survival. For panels (A) to (C) and (E), a representative random state (consistent across panels) is used to represent results.

KM analysis of predicted risk groups showed significant stratification across all 6 models with the high-risk groups showing a steep decline in survival at <2 years to ≈0.2 survival probability. From 2 to 5 years, the rate of decrease in survival probability leveled off as most high-risk patients died in the 0- to 2-year time window. In contrast to this, the low-risk group showed an approximately linear decrease in survival over the 5-year window from ≈1.0 to 0.6 (Fig. 4A). The distribution of true deaths along the ensemble score (Fig. 4B) demonstrates exceptional stratification around the tuned threshold and that the selection of a more forgiving threshold would have resulted in considerable contamination from false positives.

Each model’s continuous probability/score outputs were evaluated with univariate Cox regression. All models showed significant results, with the ensemble model performing particularly well with a c-index of 0.66 alongside a hazard ratio of 2.42 (Fig. 4C and D). This result improves against the traditional multivariate Cox approach, which, when validated against CGGA data, achieved a c-index of 0.64 and hazard ratio of 1.34 (Fig. 1H). Examining the ROC curve over time for the ensemble model (Fig. 4E) demonstrates that the model’s best performance occurs when predicting particularly severe cases with <1-year survival. In this case, the ensemble model shows an ROC–AUC of 0.86 compared to 0.68 and 0.66 for <3-year and <5-year survival, respectively.

Time-to-event information is incorporated only at the ensemble generation step; it would be relevant to consider other modeling approaches that incorporate time-to-event survival in different ways. Traditional survival approaches that do this were explored above (Fig. 1), while other machine learning methods, such as random survival forests, which implicitly utilize time-to-event data, could be a beneficial approach. Here, we emphasized the core model training as a classification problem for continuity between base models, with the ensemble step facilitating the inclusion of survival time information.

It is important to state that the expression data used for both training (TCGA) and independent evaluation (CGGA) are heterogenous and includes both WHO grade I and II patients. As a consequence, non-expression-based prognostic factors have not been controlled for; notably, IDH somatic mutation and 1p/19q codeletions would be important considerations in future approaches seeking a clinical use case. A meta-analysis of 10 LGG studies reported that the presence of the protective IDH mutation has a hazard ratio of 0.585 [35].

Training on TCGA data and evaluation against the completely distinct CGGA dataset demonstrates that the model’s performance is retained against a separate transcriptomic dataset, which will include platform and sample processing differences. However, variability in future transcriptional platform and experimental procedure should be considered at the inference stage as it is not possible to guarantee generalized performance.

### Adhesion, motility, and immune infiltration are enriched in GSL-score high-risk groups

**Fig. 5.**
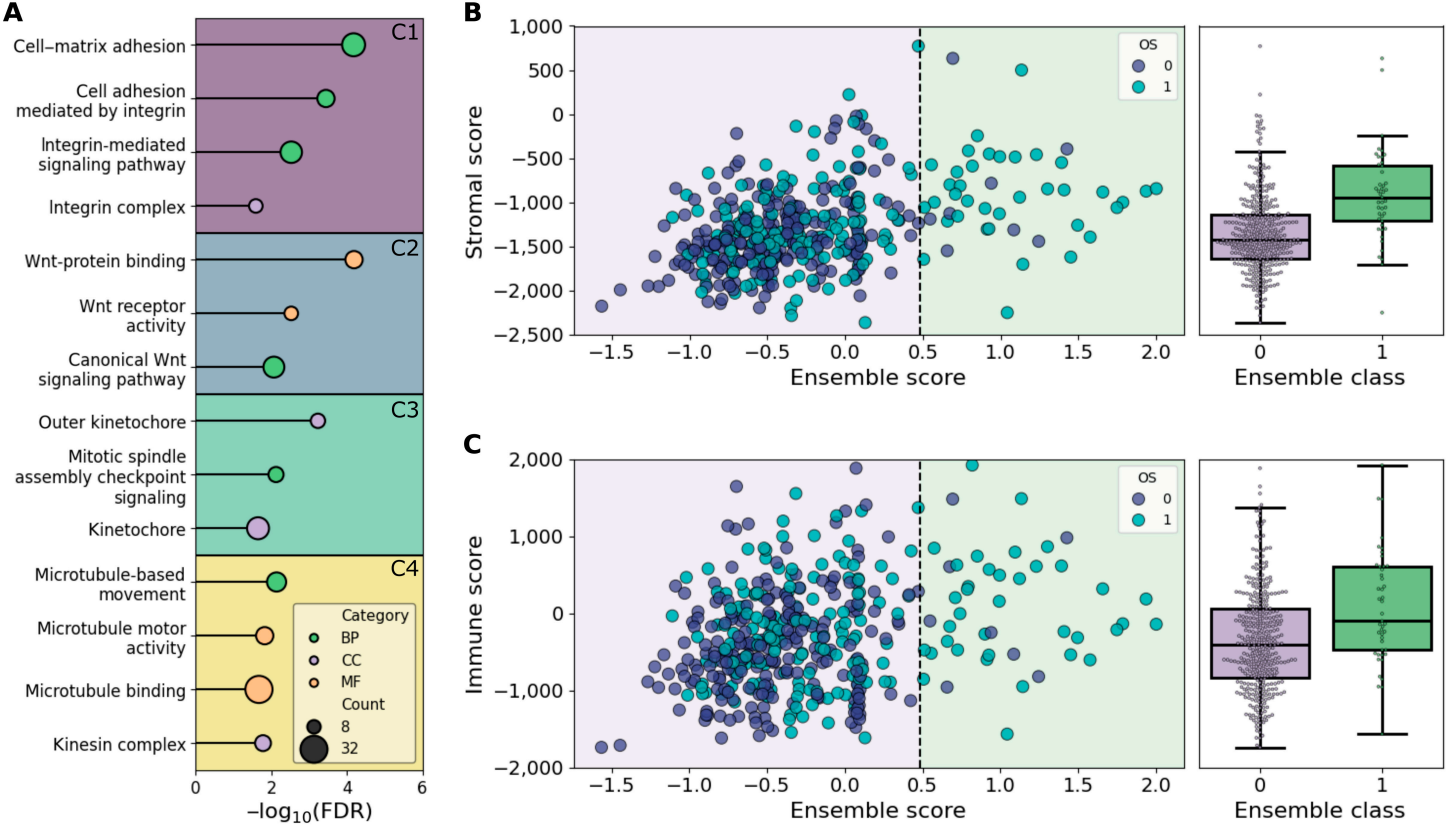
Functional analysis of model-derived risk groups. (A) Functional annotation clustering with DAVID [37] of consistently up-regulated genes in the predicted high-risk group for the ensemble model across all repeats. Clusters are labeled C1 to C4 and separated by colored boxes. Gene Ontology (GO) Direct terms are denoted by stem plot point colors. Biological Process (BP) are shown in green, Cellular Component (CC) in purple, and Molecular Function (MF) in orange. (B) Left panel: Stromal score calculated via the Estimate algorithm [52] against ensemble model scores (for a representative model). Decision threshold denoted by the dashed black line, with the low-risk region highlighted in purple and the high-risk region in green. Points are colored by overall survival, with death represented as light blue and unrecorded as dark blue. Right panel: Boxplots and points showing stromal score (linked axis to left panel) against ensemble predicted class. (C) Left panel: Immune score calculated with the Estimate algorithm [52] against ensemble model scores (for a representative model, consistent with panel (B)). Data presented as in panel (B).

To explore correlated biological pathways that may be of biological relevance, we performed differential expression analysis between high- and low-risk groups predicted by the ensemble model. We did this for each of 5 repeat experiments for the TCGA model training and CGGA validation, performed at different random states. Genes that were significantly up-regulated in every repeat were selected for subsequent Gene Ontology (GO) analysis via DAVID [36]; 1,079 genes were identified and analyzed for enrichment across GO Direct Biological Process, Cellular Component, and Molecular Function. Functional annotation clustering was performed [37] to identify more specific biological insights (Fig. 5A).

The most enriched cluster, with a cluster enrichment score of 5.12, included 4 GO terms relating to integrin signaling and cell adhesion. This observation is consistent with the well-characterized association between GSL synthesis and cell adhesion, particularly the detrimental impact of ganglioside loss in the central nervous system [38,39]. In addition to reduced cell adhesion, disruption of GSL biosynthesis can also impact integrin gene expression [40]. This previous study demonstrated that the specific shift between the production of sulfated versus sialylated GSLs has differential effects on integrin and growth factor receptor gene expression, supporting that the GSL composition of a cell fine-tunes gene expression and cell behavior. Recent work has also identified a growth-factor-triggered GSL-driven mechanism by which integrins are removed from the plasma membrane surface to mediate cell motility [41]. Together, these data support a mechanistic link between the GSL composition of a cell and integrin-mediated cell adhesion, and the correlations identified here suggest these may represent functionally relevant links between GSL gene expression changes identified here in LGG.

Three Wnt-signaling-associated genes also formed an enriched cluster (cluster enrichment score = 4.56). The activation of Wnt signaling involves the binding of Wnt proteins to their receptor complexes in ordered plasma membrane microdomains enriched with sphingolipids [42]. LRP6 is an essential coreceptor for Wnt signaling and binds the GSL GM1 within membrane microdomains [43]. Disruption of membrane order and altered GSL metabolism interfere with the phosphorylation of LRP6, disrupting downstream signaling [42]. Interestingly, the GSL mannosyl glucosylceramide promotes synaptic bouton assembly at the neuromuscular junction in *Drosophila* by binding and enhancing the levels of Wnt1/Wingless at the plasma membrane, facilitating presynaptic Wnt signaling [44]. Misregulation of Wnt signaling is linked to disrupted plasma membrane lipid organization in a range of cancers, with Wnt signaling being one of the key processes in the regulation of cell stemness, which is often co-opted in cancer [45,46]. This aligns with previous literature highlighting that *B3GNT5* gene expression increased the stem-like behavior of cells in GBM [22]. Additional studies have further implicated *B3GNT5* in tumor promotion and the stemness of a range of cancers [23,47]. Although we have not validated in LGG samples the GSL changes predicted by our gene expression analysis, these functional roles for GSLs in cancer-relevant pathways support this as an interesting avenue for future studies.

Kinetochore- and mitotic-spindle-associated GO terms were identified in cluster 3, with an enrichment score of 4.10, suggesting implications in cell division for the GSL risk group. Several enzymes involved in GSL metabolism have been shown to play important roles in cytokinesis, including GAL3ST1, ST8SIA5, and UGCG [48,49]. Individual depletion of the expression of these genes results in failure of cell division, potentially due to the mislocalization of cytoskeletal proteins that connect the plasma membrane to the actin cortex. In addition to mislocalization, altered GSL expression changes the expression of cytoskeletal genes. The depletion of *UGCG* gene expression reduced stem cell proliferation and decreased the expression of microtubule-associated protein 2 (MAP2) in neurons and glial fibrillary acidic protein (GFAP) in glial cells [50]. Potentially related to this, the fourth GO term cluster (with an enrichment score of 3.28) relates to microtubule movement, binding, and the kinesin complex. Sphingolipid-rich membrane microdomains dynamically interact with the underlying cytoskeleton regulating signaling and mediating communication across the plasma membrane [51]. Although the molecular details of this process remain poorly understood, the impact of altered GSL metabolism on the cytoskeleton in brain cancers may provide an exciting area of research.

Immune and stromal infiltration scores were calculated via the Estimate algorithm [52] and compared across GSL risk scores and risk groups (Fig. 5B and C). For both immune and stromal scores, there is a subtle increase in high-GSL-score patients. This, alongside the array of immune-related GSL functions [18], marks GSL-related immune dysfunction at tumor sites as another potential point of study for the mechanistic understanding of GSLs in disease severity [28].

### The potential for GSL-based prognostics

The effective risk stratification achieved through our ensemble machine learning approach suggests the expression and metabolic network of GSL synthetic genes carries information highly relevant to disease severity in LGG. It can be postulated that in these high-risk cases predicted with our GSL-based method, there will be dysregulated GSL synthesis and correspondingly aberrated GSL enrichment in cells. Considering the shedding of GSLs into the tumor microenvironment, with their detection in patient serum demonstrated in a number of cancers [53], GSL-based prognostics and diagnostics may represent useful future tools to identify high-risk patients. Depending on the overall downstream consequences to tumor aggression of disrupted GSL synthesis, such high-risk patients may benefit from treatment supplementation with future GSL-targeting therapies. These could potentially act to inhibit GSL synthesis or also exploit GSLs to target the cancer. Such approaches have already been exploited for neuroblastoma, where the monoclonal antibody dinutuximab binds GM2 to target cancer cells [54–56]. Tumor-associated gangliosides have also shown promise for early-stage detection in ovarian cancer [57].

With these models being trained on limited, open-source data, the potential for further development with more extensive clinical data is considerable. This is particularly relevant as society moves toward precision medicine approaches, where these types of methods could provide guidance for specific treatment strategies on a patient-by-patient basis. This GSL-specific approach could easily be multiplexed alongside equivalent methods focusing on different metabolic pathways, giving patients biologically relevant risk stratification to assist clinical decision-making.

A recent systematic review of glioma prognostic studies demonstrates that most approaches derive risk scores directly from transcriptomic signatures using linear modeling frameworks applied to large cohort datasets [58]. Our approach uses the same underlying transcriptomic data but supplements them with pathway-informed metabolic modeling of GSL biosynthesis. This positions our approach alongside existing transcriptomic signature approaches while extending them to incorporate network-level metabolic context and multimodel prediction.

The relevance of changes in GSL biology to patient outcomes in LGG, demonstrated here, suggests new avenues for investigation to understand mechanisms driving disease severity. This should be considered with the caveat that the changes to GSL synthetic gene expression may be consequences of general metabolic reprogramming in severe LGG. Attempts to decouple this potential confounder will require extensive experimental work to establish if GSL metabolism is a direct driver of disease severity.

## Methods

### Data accession

Training data for LGG (grades III and IV) were accessed from the TCGA via the Xena database [59]. Transcripts per million (TPM) gene expression data for GSL synthetic enzymes were acquired from the TCGA–TARGET–GTEx dataset hosted on Xena [59,60]. Rows with missing values for GSL synthetic enzyme expression were removed.

Raw count data and metadata for both LGG and GBM were also accessed from the TCGA via the Xena database for differential expression analysis.

Validation data were accessed from the CGGA [61] as fragments per kilobase of transcript per million mapped reads (FPKM) values, which were converted to TPM values and log_2_(*x* + 0.01) normalized before filtering to the relevant GSL synthetic genes to match the training data format.

The TCGA LGG cohort included 523 patients. The CGGA LGG cohort included 443 patients. The clinical variables included were overall survival and overall survival time.

The GSL synthetic enzyme genes included for model development were as follows: *A4GALT*, *ABO*, *B3GALNT1*, *B3GALT1*, *B3GALT4*, *B3GALT5*, *B3GNT2*, *B3GNT5*, *B4GALNT1*, *B4GALT5*, *B4GALT6*, *FUT1*, *FUT2*, *FUT3*, *FUT5*, *FUT6*, *FUT9*, *GAL3ST1*, *GCNT2*, *ST3GAL1*, *ST3GAL2*, *ST3GAL3*, *ST3GAL4*, *ST3GAL5*, *ST3GAL6*, *ST6GALNAC2*, *ST6GALNAC3*, *ST6GALNAC4*, *ST6GALNAC5*, *ST6GALNAC6*, *ST8SIA1*, *ST8SIA5*, *UGCG*, and *UGT8*.

### Differential expression analysis

All differential expression analyses were performed in R using the DESEQ2 package [62]. Raw count data were used, with low-count genes filtered out before analysis. Complete scripts for the differential expression analysis are available on GitHub (https://github.com/JackWJW/LGG_Prognosis_Prediction).

### Survival analysis

KM analysis was conducted in the lifelines package in Python [63].

Elastic net regression analyses were performed with the scikit-survival package in Python [64]. Full lasso regression was performed with scikit survival L1 regularization set at 0.5.

Cox regression analysis of GSL synthetic enzymes from the TCGA data was performed with the lifelines package in Python, utilizing the best alpha (0.05) identified from lasso/elastic net regression (0.5 L1 regularization) experiments; 0.05 was utilized as the L2 regularization for subsequent ridge Cox regression analyses performed against genes and models.

The 4-gene signature was calculated by performing multivariate regression against the 4 key genes identified from the lasso regression feature selection experiment and subsequently weighting expression values for each gene by their Cox coefficients to calculate their aggregate risk score (Eq. (1)).

$$\text{Risk score}=\sum_{g}^{n}E_{g}C_{g}$$ (1)

where *E_g_* refers to a gene’s expression and *C_g_* refers to a gene’s Cox coefficient.

### Metabolic modeling and simulations

Metabolic modeling was performed using the custom-written pyGSLModel Python package. pyGSLModel was built on top of COBRApy [31], iMATpy [33] (https://pypi.org/project/imatpy/), and pyfastcore [32] (https://pypi.org/project/pyfastcore/) to simplify the preparation of sphingolipid-metabolism-specific models and subsequent transcriptomic integration and metabolic simulation. A sphingolipid-specific metabolic model was derived from the human-genome-scale HUMAN-GEM model [34]. Trial simulations were run on HUMAN-GEM with a neuronal GSL-specific objective function defined based on key GSL proportions known in neuronal cell models [65]. Reactions carrying flux in trial simulations as well as manually curated reactions affiliated with sphingolipid catabolism were defined as core reactions before performing automated model pruning of HUMAN-GEM with pyGSLModel utilizing pyfastcore. This process resulted in a specific model of GSL synthesis.

Transcriptomic integration was performed via the iMAT method, which was implemented via iMATpy in pyGSLModel. With respect to training TCGA data, iMAT simulations were performed for each LGG sample in the dataset, leveraging 34 genes of GSL synthesis to define iMAT rankings. The pruned metabolic model of GSL synthesis was utilized for all iMAT simulations. To enable the identification of the best-performing simulation parameters, 24 different parameter sets were trialed. Each parameter set includes variable values for the upper quantile (% of highest-expressing genes to be denoted +1 for iMAT), lower quantile (% of lowest-expressing genes to be denoted −1 for iMAT), threshold (flux above which the solution score is increased for +1 reactions), and epsilon (flux above which the solution score is penalized for −1 reactions). Parameter sets are shown in Table 1.

The list of lipid-producing reactions included in model training datasets is as follows: d-galactosyl-*N*-acylsphingosine, GA1, GA2, GD1a, GD1b, GD1c, GD2, GD3, GM1, GM1b, GM2, GM3, GQ1b, GQ1c, GT1a, GT1b, GT1c, GT2, GT3, fucosyl-galactosylgloboside, galactosylgloboside, globoside, globotriaosylceramide, lactoneotetraosylceramide, lc3Cer, lc4Cer, sialyl-3-paragloboside, sialyl-galactosylgloboside, sulfatide, galactocerebroside, type I H glycolipid, and type II H glycolipid.

The lipid series whose total fluxes were included in model training datasets are as follows: (neo)lacto-series, 0-series(ganglio), a-series(ganglio), b-series(ganglio), c-series(ganglio), gal-series, and globo-series.

CGGA data for GSL synthetic genes underwent iMAT simulation with parameters from the best-performing parameter set (identified in TCGA cross-validation).

pyGSLModel iMAT integration functions also calculated total fluxes throughout lipid series as well as individual fluxes for reactions of GSL synthesis. These total lipid-series fluxes were also included in the simulated flux appended datasets for model training and validation.

### TCGA model cross-validation

TCGA-only models were trained under nested cross-validation with 3 stratified outer and inner folds. In the data preparation pipeline, data underwent *z*-score normalization (sklearn) alongside SMOTE oversampling (imblearn). All models underwent 25 iterations of Bayesian optimization (BayesSearchCV, sklearn) in the inner CV loop, with scoring on average precision (area under the precision–recall curve). Outer fold cross-validation results were pooled for evaluation. This process was repeated for each different set of iMAT parameters, at 5 different random states (0 to 4). SVM (sklearn), RF (sklearn), XGB (XGBoost), LR (sklearn), and ANN (pytorch, skorch) models were trained with this approach. The ANN was trained for a maximum of 300 epochs with early stopping, focal loss, and the Adam optimizer [66–69]. The ensemble prediction was generated by performing multivariate Cox regression of the 5 models’ probabilities for validation samples against overall survival and survival time. The resulting Cox coefficients were utilized as weights for ensemble model calculation (Eq. (2)).

$$\text{Ensemble score}=\sum_{m}^{n}p_{m}C_{m}$$ (2)

where *p_m_* refers to model probability and *C_m_* refers to the model Cox coefficient.

### Final model training (TCGA data) and evaluation (CGGA data)

The final model was trained on all TCGA data with Bayesian optimization performed under 5-fold cross-validation for 50 iterations with scoring on average precision. In the data preparation pipeline, data underwent standard scaling (sklearn) alongside SMOTE oversampling (imblearn). This process was repeated across 5 different random states (0 to 4). SVM, RF, XGB, LR, and ANN models were trained with this approach. The ANN was trained for a maximum of 300 epochs with early stopping, focal loss, and the Adam optimizer. The ensemble model generated its prediction as previously, via multivariate Cox regression of model probabilities to generate Cox coefficients to calculate a Cox-weighted ensemble score (Eq. (2)).

The resulting models were validated against CGGA data.

### GO analysis

Differential expression analysis was performed between high-risk and low-risk groups predicted by the ensemble model against CGGA data. This was done for each of the 5 repeats, selecting genes that were consistently up-regulated in all repeats to take forward for GO analysis.

GO analysis was performed using the DAVID web server [37] against GO Direct terms for Biological Process, Cellular Component, and Molecular Function. Functional annotation clustering was performed on the “Medium” Classification Stringency with an EASE threshold of 1. Results from clustering were filtered for cluster enrichment scores >3.0 and Benjamini–Hochberg-adjusted *P* values ≤0.05.

### Tumor purity analysis

Tumor purity, immune, stromal, and Estimate scores were calculated using the Estimate algorithm (tidyestimate package in R) against CGGA FPKM data for all genes and samples.

### Data visualization

Differential expression volcano plots were generated with the ggplot package in R [70]. All other data visualization was performed using the matplotlib and seaborn packages in Python [71,72].

## Data Availability

All data, code, and models are available on GitHub (https://github.com/JackWJW/LGG_Prognosis_Prediction) and Hugging Face (https://huggingface.co/JackWJW/LGG_Prognosis_Ensemble). The pyGSLModel Python package is available on pypi (https://pypi.org/project/pyGSLModel/), with the complete code on GitHub (https://github.com/JackWJW/pyGSLModel), providing functionality for GSL-specific metabolic simulations and calculating GSL risk scores from RNA sequencing data. Package development and analysis was performed in Python 3.11. Model training was performed on a commercial graphics processing unit.
